# Catching met- and unmet care needs in real-world catchment area: the Antwerp experience

**DOI:** 10.1192/j.eurpsy.2025.157

**Published:** 2025-08-26

**Authors:** G. Dom

**Affiliations:** University of Antwerp, Antwerp, Belgium

## Abstract

**Abstract:**

Increasingly the collection and use of clinical data is considered as extremely important. These data may allow, among other targets, a better profiling of patients and as such help to develop better and more targeted care-pathways. Often these type of data collections are implemented on large, national levels. Although this already provides an important source of information, often the regional specifics are missed on these larger scales. Within the Antwerp region we developed a program allowing a deeper, smaller grained, level analyses of populations mental health care needs and their regional differences. The aim is to use these data to steer changes in the care pathways as offered by the different locally active care-providers.

**Disclosure of Interest:**

None Declared

